# A Case Report on Prepubertal Vaginal Bleeding due to Leech Bite: History Is Very Important

**DOI:** 10.1155/2021/8896600

**Published:** 2021-02-23

**Authors:** S. M. Tajdit Rahman, Tasnim Shahriar, Adiba Tarannum, Md Abul Kasem, Tasnuva Akter

**Affiliations:** ^1^UHC, Baliadangi, Thakurgaon, Bangladesh; ^2^National Institute of Diseases of the Chest & Hospital, Dhaka, Bangladesh; ^3^Khulna Medical College, Khulna, Bangladesh; ^4^Green Life Medical College, Dhaka, Bangladesh; ^5^Bikrampur Bhuiyan Medical College, Bangladesh

## Abstract

Though prepubertal vaginal bleeding is rare, it can create a dilemma for both physicians and parents. Prepubertal vaginal bleeding due to leech bite is even rarer and very difficult to diagnose without proper history. We report a case of a six-year-old girl presenting with vaginal bleeding and ultimately diagnosed as a case of vaginal bleeding due to leech bite. We described how focused history helped us in diagnosis and management of such case in rural areas with limited healthcare resources. A detailed history with a high index of suspicion and a thorough examination is essential to diagnose leech bite in the vagina.

## 1. Introduction

Prepubertal vaginal bleeding is a worrisome condition for both the parents and child and the physician as well. Though prepubertal vaginal bleeding is rare, it has multiple etiology, including sexual abuse, local trauma, foreign bodies, infection, precocious puberty, hemangioma, and tumors [1, 2]. Vaginal bleeding due leech bite is even rarer, and there are very few reported cases; most of them are from tropical regions [2, 3]. Though Bangladesh is a tropical country, only a few prepubertal vaginal bleeding cases because of leech bite have been reported [4].

Here, we describe a case report of a 6-year-old girl who came to the primary care emergency setting of rural Bangladesh with a short history of unexplained vaginal bleeding after swimming and ultimately diagnosed as a case of vaginal bleeding due to a leech bite.

## 2. Case Report

Our patient, a six-year-old girl, was taken by her parents to the Upazila Health Complex's emergency setup, which is the rural primary health care center of Bangladesh with per vaginal bleeding for the last 2 hours. According to the child's mother, bleeding was mild at the beginning, but it increased with time. There was no other bleeding site, and she had no history of obvious trauma or sexual abuse. The patient did not have any significant past history. Her vaccination was complete according to the EPI (Expanded Program on Immunization) schedule of Bangladesh. She was a bit anxious and pale. Her blood pressure was 95/65 mm Hg with a pulse rate of 118 per minute, and respiratory rate was 30 per minute. She had an average body weight for her height.

On examination, she looked pale, and her abdomen was normal. Her perineal area was packed with several pieces of cloth that were soaked with blood. After removing the pieces of cloth, there was active bleeding from external genitalia. Her hymen was intact, and there were no signs of injury in the external genitalia. Through perineal examination of the patient was not possible at that time because she was agitated. An external pack was given by a sanitary napkin, and the patient was transferred immediately to OR after fluid resuscitation. Meanwhile, further questioning, the parents revealed that she had a history of swimming in a nearby pond just before the incident, which allows us to consider leech bite as a differential diagnosis.

Later on, the patient's perineal area was examined thoroughly in the OR under deep sedation. We explored the perineal area, and it was negative for any injury or lacerations or signs of trauma. There was bright red blood and clots over the introitus. After removing the clots and blood, it was evident that bleeding was coming from the vagina. Vaginoscopy was not performed in the first attempt with a view to avoiding unnecessary injury to the hymen. The vagina was widened and exposed with finger, which revealed several pieces of clots. After removing the clots with mosquito forceps, a moving black object was seen along the right lateral wall of the vagina. About 200 ml normal saline was irrigated into the vagina, which facilitates dislodgement of 1.5 cm leech from the vagina to the introitus. Then, it was removed gently by forceps. After that, a mucosal injury was found along the right lateral wall just above the external vaginal opening and which seems to be the site of active bleeding. No photo was taken of the external genitalia because of the parent's religious belief.

The vagina was further flushed with 100 ml normal saline, and iodine-soaked gauze was used as an internal vaginal pack. The patient was transferred to the inpatient department with a diaper, and her diaper was checked every 30 minutes. There was no more bleeding, and the internal pack was removed after 12 hours. Further examination of external genitalia showed no active bleeding, and the patient was stable. She was discharged after 24 hours of her admission with an oral antibiotic. After seven days of follow-up, we did not find any signs of infection or complication.

## 3. Discussion

Leeches are hematophagous worms with segmented bodies and are parasitic to man and other animals [5]. Leeches belong to the phylum Annelida, class Hirudinea [6]. They can suck and usually store blood up to multiple times of its body size, and the host is usually unaware of this attachment due to the presence of the anesthetic agent in the leech saliva [7]. Common site of leech infestation is the skin whereas leech can be found in body cavities such as the nasal cavity, pharynx, larynx, trachea, esophagus, urinary bladder, rectum, and vagina [3, 6].

Vaginal bleeding due to leech bite is rare and can cause various complications in the human body, which may vary from minor bleeding to severe conditions like severe anemia and profound hypovolemic shock [8, 9]. The complications arise from leech bite mainly due to either mechanical obstruction of vital organs and/or bleeding [6, 10]. One of the main morbidities due to leech bite, hemorrhage, is not because of the sucking of blood by the leech but due to the presence of various substances in leech saliva that include hirudin, theromin, bufrudin, granulin-like peptide, and haemadin [11, 12].

Publications on leech infestations in the vagina are limited, with most of those are case reports. We found a single study from Bangladesh regarding leech infestation in children through body orifices, which reported few vaginal bleeding cases in children due to leech bite [4]. Vaginal bleeding before puberty in an apparently healthy girl could make a troublesome situation for the physician, especially without proper history. Hannan et al. reported 9 of 17 cases reported with a history of leech entry and doubtful history of its exit [4].

Most of the reported cases of vaginal leech bites are from tropical and semitropical areas and occurred after swimming or bathing in freshwater [3]. Careful and detailed history can guide to suspect leech bite in prepubertal patients from the tropical region with vaginal bleeding. Details and focused history and clinical examination of the patient are the mainstays of diagnosis in case of bleeding due to leech bite.

The management of leech infestation is focused on two parts [3, 6, 12]. One of them is supporting treatment at the presentation to minimize the complications that arise from the leech bite, and another is the removal of the leech from the site [10]. Supporting treatment may consist of intravenous crystalloids, blood transfusion, and iron sulfate [13]. The most typical, easiest, and safest way is to apply normal saline as done in most reported cases of the literature [9, 14]. But other agents like salt, alcohol, or vinegar have been used to remove leech or local anesthetic agents like lidocaine is being applied to paralyze the parasite. Removal of the leech should not be done forcefully because parts of the leech may remain in the wound, which will lead to continuous bleeding and infection in the later period [5, 14]. If bleeding is stopped after a short period, no further treatment is required. If bleeding continues, the vagina should be examined, and the bleeding site should be thoroughly washed with normal saline to ensure that leech saliva is completely removed. The vagina should be packed with pressure gauze after complete removal of the parasite, and the gauze must be removed after 24 hours [15, 16]. In the case of vaginal leech infestations, the leech usually remains in the vagina, but cases were reported where leech migrates to the uterus and peritoneal cavity that can lead to catastrophic complications [17, 18].

## 4. Conclusion

Despite its rarity, prepubertal vaginal bleeding due to leech bite should be suspected, especially if the patient from a tropical region has a history of using the pond and/or river water for bathing to decrease morbidities and complications from diagnostic failure or delay. A focused and detailed history with a high index of suspicion and a thorough examination is essential to diagnose leech bite in the vagina. Careful removal of a leech with surgical forceps is also necessary to prevent further injury.

## Data Availability

The data used to support the findings of this study are available from the corresponding author upon request.
